# Book Reviews

**Published:** 1830-08

**Authors:** 


### CRITICAL ANALYSES.

Quæ laudanda forent, et quæ culpanda, vicissim

Illa, prius, cretâ; mox hæc, carbone, *notamus*.—PERSIUS.

*The unnoticed Theories of* SERVETUS. By GEO. SIGMOND, M.D. F.L.S. 2d Edition.—8vo. pp. 135. London, 1828.

DR. SIGMOND is so fortunate as to possess a copy of the "Christianismi Restitutio" of Servetus, bequeathed to him by the late Dr. SIMS, who prefixed the following note to it.

"The fate of this work has been not a little singular: all the copies, except one, were burned along with the author, by the implacable Calvin. This copy was secreted, and saved by D. Colladon, one of the judges. After passing through the library of the landgrave of Hesse Cassel, it came into the hands of Dr. Mead, who endeavoured to give a quarto edition of it; but, before it was nearly completed, it was seized by John Kent, messenger of the press, and William Squire, messenger in ordinary, on the 27th of May, 1723, at the instance of Dr. Gibson, bishop of London, and burnt, a very few copies excepted. The late Duke de Valliere gave near 400 guineas for this copy, and at his sale it brought 3810 livres. It contains the first account of the circulation of the blood, above seventy years before the immortal Harvey published his discovery."

On this Dr. Sigmond says, oddly enough,

"In justice to the memory of my late valued friend, I must state my conviction that this copy is not the original one: at the same time, I firmly believe he imagined it to be that which he has described."

The progress of toleration, though slow, is sure: three centuries ago the same flames devoured the author and his work; in 1723 the book was burnt, but Dr. Mead was spared; and in the present age the whole of Servetus might be republished, without fear of bishop or reviewer: we are, therefore, something discontented with Dr. Sigmond for giving us only forty pages of the unitarian physician, and filling up the rest of his book with heaps of introductory matter, and an inaugural dissertation on the Bath waters, which is smuggled in at the end, without being *declared* on the titlepage.

The first extract from Servetus is a prayer, p. 46-48.

The second extract, from p. 54 to 78, contains an account of the three spirits which are in the body. The first is the blood, whose seat is in the liver and the veins. The second is the vital spirit, whose seat is in the heart and arteries. The third is the animal spirit, resembling a ray of light, whose seat is in the brain and nerves. Life begins in the heart, which takes its materials from the liver, but in return bestows life upon it. Life is composed of the blood from the liver, by a wonderful process, to be explained presently, and therefore life is said to be in the blood by God himself; Gen. ix. Levit. xvii. and Deut. xii. Now, the vital spirit is formed from the air which is inspired, and the finest part of the blood. The vital spirit takes its origin from the left ventricle of the heart, the lungs bearing a great part in its production. It is a thin spirit, formed by the force of heat, of a scarlet colour, and fiery power, being a kind of bright vapour from the purest blood, and containing in itself the substance of water, air, and fire. It is generated by the mixture which takes place in the lungs, of the inspired air with the refined blood sent from the right to the left ventricle. But this transmission does not take place through the septum cordis, as is vulgarly supposed, but the refined blood is very artificially drawn from the right ventricle by a long passage through the lungs: it is prepared and made scarlet* by the lungs, and it is transmitted from the pulmonary artery into the pulmonary vein. It is then, in the pulmonary vein, mixed with the inspired air, and is cleansed from its soot by expiration.

That this communication and preparation takes place by means of the lungs, we learn from the communication and joining of the pulmonary artery and vein in the lungs. This is confirmed also by the great size of the pulmonary artery, which would not be so large, nor convey so much blood to the lungs, solely for their nourishment; particularly as the lungs of the fœtus derive their nourishment from another source, the valves of the heart not being open till birth, as we are taught by Galen. Nor is simple air sent from the lungs to the heart, but air mixt with blood, through the pulmonary vein: therefore the mixture takes place in the lungs. The spirituous blood derives its scarlet colour from the lungs, and not from the heart. There is not sufficient room for so great a mixture in the left ventricle, nor for the elaboration necessary to form the scarlet colour. Lastly, the septum cordis, being deficient in vessels and power, would be unfit for this communication and elaboration, though something may drain through it. By the same artifice by which there is a transfusion in the liver from the vena porta to the vena cava, on account of the blood, there

* We translate flavus *scarlet*, as Servetus could not have mistaken the colour of arterial blood, though he might the meaning of *flavus*.

is also a transfusion in the lungs from the pulmonary artery to the pulmonary vein, on account of the spirit.

Such were the discoveries of the great Servetus, and so nearly had he fixed upon his own head the never-fading wreath that crowns the brows of Harvey!

"He then commences," says Dr. Sigmond, "that most extraordinary passage upon the seat of the mind. The blood, he supposes, having received, in its passage through the lungs, the breath of life, is sent by the left ventricle into the arteries; the purest part ascends to the base of the brain, where it is more refined, especially in the retiform plexus. It is still more perfected in the small vessels, the capillary arteries, and the choroid plexus, which penetrate every part of the brain, enter into the ventricles, and closely surround the origin of the nerves. From the vital spirit it is now changed into the animal spirit, and acts upon the mass of brain, which is incapable of reasoning without this stimulus. In the two ventricles of the brain is placed the power of receiving impressions from external objects; in the third, is that of reasoning upon them; in the fourth, is that of remembering them. From the communication through the foramina of the ethmoid bone, the two ventricles receive a portion of external air to refresh the spirit, and to give new animation to the soul. If these ventricles are oppressed by the introduction of noxious vapour, epilepsy is produced; if a fluid presses on the choroid plexus, apoplexy; and whatever affects this part of the brain causes loss of mental power." (P. 14, 15.)

The next extract, from p. 79 to 86, treats of animal and vegetable life. Servetus is of opinion that the soul is breathed into the fœtus at the moment of birth, and that the fœtus, breaking its membranes before it actually lives, is to be compared to a chicken breaking its shell, or to those plants which tear asunder walls and rocks by their growth.

The last extract, from page 86 to 90, which is taken from the book De Fide et Justitiâ, treats of the partition of the mental faculties between the heart and brain. Faith and the will reside in the former, the power of reasoning in the latter.

Before we take leave of Dr. Sigmond, we must observe that there are more blunders of the press, or the pen, in this little work, than we should have expected, and it is not always easy to know to which to attribute them. The last sentence in Servetus's prayer (page 48) is turned into nonsense, by *foco* usurping the place of *fuco*. In the next page the Doctor quotes the well-known verse of Lucretius,

Tantum religio potuit suadere malorum, so as to make it a verse no longer,

"Tanta potuit religio suadere malorum."

These mistakes, however, together with some others, are easy to correct; but the following passage has altogether baffled our critical sagacity, "Illo die ducebatur illum impia nos rationis inire elementa." P. 46.

Nor would we undertake to find the following morsel of Greek in Athenæus, to whom our author attributes it at page 104, Εσθλον *αλεξ ικανον πασῃ ανιῃ*. Perhaps it should be Εσθλον *αλεξικακον πασῃ ανιῃ*.

In conclusion, we thank Dr. Sigmond for presenting us with a considerable portion of Servetus in an accessible shape, and hope that, in his third edition, he will give us larger extracts, and leave the Bath waters to the Bath Guide.

---

*An Outline of the first Principles of Botany.* By JOHN LINDLEY, F.R.S. L.S. and G.S. &c. Professor of Botany in the University of London.—Pp. 106. Longman and Co. 1830.

*A Synopsis of the British Flora, arranged according to the natural Orders: containing Vasculares, or flowering Plants.* By JOHN LINDLEY, F.R S. L.S. and G.S., Member of the Imperial Academy Naturæ Curiosorum of Bonn; of the Botanical Society of Ratisbon, and of the Physiographical Society of Lund; corresponding Member of the Linnæan Society of Paris; assistant Secretary of the Horticultural Society; and Professor of Botany in the University of London.—Pp. 360. Longman, 1829.

*A Natural Arrangement of British Plants, according to their relations to each other, as pointed out by Jussieu, De Candolle, Brown, &c., including those cultivated for use: with an Introduction to Botany, in which the Terms newly introduced are explained; illustrated by Figures.* By SAMUEL FREDERICK GRAY, Lecturer on Botany, the Materia Medica, and Pharmaceutic Chemistry.—Two vols. pp. 824 and 760; twenty-one Plates. Baldwin and Co. 1821.

THE works of GRAY and LINDLEY, though published at such distant dates, are very similar, both in their objects and contents. They are, as their titles predicate, introductions to, and synopses of, the so called natural method of botanical arrangement, which has long emulated, and now attempts to supersede, the Linnæan system. From them we may consequently learn the progress which the cause they advocate has made during the last ten years. The later published, of course, contain allusions to the more modern discoveries in botany; but the former, notwithstanding its manifold defects, still remains the most complete introduction to the natural system that has yet appeared in the English language; for it not only includes the British Cryptogamic as well as the Phænogamic Flora, but also comprises many of the plants most commonly cultivated here, whether in the garden, the forest, or the field. The great objection to the work of Gray, and the cause of its very general neglect, is the unhappy passion therein evinced for changing the names of plants, and which is too often indulged for no other reason than the love of change. This is a privilege granted with jealousy, and granted but to few: it is a power that should be used with the utmost caution, and is wisely watched with the extremest care.

The first volume of Gray's work contains a copious introduction of nearly three hundred pages, explanatory of modern discoveries and modern nomenclature, designed to answer the same purpose as Lindley's shorter "Outline of the First Principles of Botany;" to which it is in some respects superior, although in others far less to be relied on. The remainder of the first volume is dedicated to the systematic arrangement of the supposed truly cellular plants, i. e. the Cryptogamic vegetables of Linnæus, excluding the ferns; and these are designed by Lindley to form the subject of the second volume of his Synopsis, as yet unpublished. Gray's second volume contains, besides the ferns, the whole of the native and the most commonly cultivated plants of Britain: a shorter view of the indigenous part of which will be found in Lindley's published volume.

We propose to show from these, two of the most strenuous advocates of the *natural* scheme, the insufficiency of its principles as a diagnosis, and the great inferiority of its artificial helpmates to the Linnæan clavis; and we shall direct our attention the more particularly to the Synopsis of Lindley, not only because he appears the most sanguine proselyte, but also because, being the latest, his work may be presumed to contain the most approved, the most perfect application of the system. A slight view, however, in the first place, must be taken of his "Outlines."

The sententious form in which this volume has been written, will in a great measure preclude remark. It chiefly consists of a series of enunciations, which we presume are fully descanted on by the Professor in his lectures; and, although far too succinct to convey much information to the uninitiated, it is a kind of classbook of which we highly approve, as it is well fitted to recal facts which might otherwise be easily forgotten, and cannot fail to assist students in the, at first very difficult, task of connecting an extended series of facts, and properly arranging the knowledge they have gained.

Many of the doctrines are, however, advanced far too dogmatically, and some things stated as absolute facts which should rather have been ventured as doubtful hypotheses: e. g. in par. 25, it is said of the spiral vessels, "they are not found in any part which is formed in a downward direction, and are consequently absent from the wood, bark, and root." Whereas Sprengel writes, "they are found also in the root, as well as in the stalk. Richard observes, "they are found sometimes in the root," and adds, "Messrs. Link and Treviranus have discovered these vessels in the roots of several plants;" and "still more recently M. Amici has unfolded spirals in the roots of many plants, and, among others, of the Agapanthus umbellatus, and of the Crinum erubescens."

Again, par. 27, "they only exist in plants propagated by the agency of sexes." Whereas Sprengel says, "We find an instance of this form (the primitive spiral,) in some Confervæ, in some of the Musci hepatici, and especially in the cellular texture which covers the surface of the Sphagnum obtusifolium." Keiser likewise supposes the spiral to be the original of all the other varieties of vegetable vessels, which he asserts are merely changes of the spiral; and this opinion (writes a contemporary of no mean credit) receives considerable support from the fact that spiral vessels are found in all young plants, and that they are the only kind observed in some of the *inferior* tribes of vegetables." We do not presume to say that Lindley is wrong, for we have never seen spiral vessels in the Confervæ or Musci, yet, when such authorities vouch for their existence, we should have been better pleased to have found the negative more modestly asserted; and, as it has been elsewhere, a "*supposed*" might here have been very advantageously introduced.

Par. 30. Fausses trachées: "sometimes they have the appearance of spiral vessels, from which they are known by not being capable of unrolling." It should rather have been, by their *greater difficulty* of unrolling; for they may be, and have been, by care, frequently unrolled.

Par. 34. "The mode in which the different forms of tissue are developed, is unknown." True: the mode of developement is not certainly known, but the observations are at least as conclusive on this point, and the hypothesis at least as reasonable, as that so positively stated with regard to the spiral vessels.

Par. 42. "The mass of cellular tissue lying *beneath the* CUTICLE of the bark is called the EPIDERMIS:" very improperly, and we believe very seldom, and by very few; for the cuticle is the *epidermis*, and the layer of cellular matter beneath the cuticle is called the *cellular integument*, the stratum cellulosum, or the medulla corticalis. Again, in par. 105, this error is repeated, and the names of two very distinct and different structures used as synonymes to designate the same: thus, "the exterior cellular tissue constitutes the *cellular integument* OR *epidermis*."

Par. 64. Of the root we read, that "anatomically it differs from the stem in the absence of *spiral vessels,* of *pith*, and of buds, and in the want of stomata." The first of these peculiarities we have already shown to be at least apocryphal, and the second must be confessed to be a controverted point, since Richard writes "this difference appears to us of little importance, and even in direct opposition to facts. In truth, we have found, in a great number of vegetables, that the medullary canal of the stem is continued, without any interruption, into the body of the root. If, for example, the trunk and root of a horse-chesnut, of two years old, be slit longitudinally, the medullary canal of the stem will be seen *to extend to the lowest part of the root.* It is the same if we examine a young plant of sycamore or maple. But very often this canal, which was very manifest in the plant shortly after its germination, *at last diminishes*, and *even gradually disappears*, in the progress of vegetation, so that it is no longer found in those adult plants which contained it when young. From this it follows that the absence of medullary canal in the root *cannot* be reckoned a distinctive anatomical character between it and the stem, because it almost invariably exists in the germinating radicle of the seed, and often in the root of a great number of vegetables, long after this first period of their life."

Par. 82, 83. "The pith: it *never* alters in diameter after it is once formed." This again is "land debateable:" we strongly suspect that it lessens gradually in diameter, and in old stems and branches becomes entirely obliterated; and, notwithstanding Du Petit Thouar's assertion, we have specimens which we think prove this gradual diminution of the medullary canal. This opinion receives confirmation from the extract just made from Clinton's Richard.

Par. 103 and 104, the phrase "woody fibre" should be changed for "bark fibre," as it is likely to lead to confusion of ideas when we read that "the *woody* fibre and ducts constitute the *liber*."

Par. 138 and 139. "Whatever is produced by the evolution of a leaf-bud is a branch." "A spine (i. e. a thorn,) is the imperfect evolution of a leaf-bud, and is therefore a branch." Notwithstanding the force of this syllogism, we must still contend that a spine is not a branch. Leaf-buds often evolve nothing but leaves, as in the firs and their allies: leaf-buds often evolve tendrils only, and tendrils and leaves conjointly are often produced by the evolution of a leaf-bud, but neither leaves nor tendrils are *branches:* a branch is the elongated axis of a leaf-bud, combined with its appendages, but the axis often becomes abortive, and the appendages only are evolved; yet the appendages alone do not constitute the branch, any more than the imperfect evolution of the abortive axis. But we suppose that it is to the partial, rather than to the imperfect, evolution of a leaf-bud that reference here is made; in which the developement becomes suddenly arrested, so that a short branch is produced terminating in a spine or thorn : but the branch which bears the thorn, (often several thorns,) is not itself the thorn, any more than a root which bears a bulb is a bulbous root. Contrast, for instance, the spines of the cockspur-thorn with the thorn-bearing branches of the hawthorn. Hence, it should rather have been said, "A spine is the imperfect evolution of a leaf-bud, and *therefore* it is NOT a branch." This needless paradox reminds us of the schoolboy's sophism, "No cat has two tails; a cat has one tail more than no cat: ergo, a cat has three tails;" or, to use more parallel terms, "Whatever time results from the revolution of the earth round the sun is a year; a week is an imperfect or partial revolution of the earth round the sun; therefore, a week is a year."

Par. 299. Whatever intervenes between the bracteæ and the stamens belongs to the floral envelopes, and is either calyx or corolla; of which nature are many of the organs VULGARLY called *nectaries*." This is a gratuitous assumption, apparently introduced for the purpose of excluding a very convenient Linnæan term. It is a fact well known to all that the various forms of appendage found on the vegetable frame, such as scale, leaf, bracte, sepal, petal, stamen, pistil, seed-vessel, &c., are all but stages of developement of the same rudimentary organizations, and that, as circumstances favor, either the one or the other may be predominantly evolved. But as each becomes successively and variously unfolded, it has been found convenient to give to each form or stage of metamorphosis an appropriate and peculiar name; and, if *calyx* be allowed to designate one form, and *corolla* another, *nectary* may surely be permitted to denote a different (often an intermediate) series of evolutions; and, although a far too great latitude has been occasionally indulged in, the transitional developements are more conveniently associated and indicated thus, than by calling them abortive organs of various kinds, when it is very doubtful what organs they are abortions of, or if, in truth, many of them are abortions at all.

The definitions of the several modes of inflorescence are exceedingly unsatisfactory, in some instances, we think, incorrect; or, at any rate, to us unintelligible. E. g. par. 265 and 267 declare that "a *panicle* is a raceme, the flower buds of which have, in elongating, developed other flower buds." "A panicle, the elongation of all the ramifications of which is arrested, so that it assumes the appearance of an umbel, is called a *cyme*." Now, the essence of a panicle is, that the partial peduncles should arise from different points of the common peduncle, as do the pedicles of a raceme, and as do the sessile or subsessile flowers of a spike; but the essence of a cyme is, that the first divisions all spring from the *same* point, and that the subdivisions only be irregular. Now, if a panicle which is defined as being a *raceme*, the flower buds of which, in elongating, develope other flower buds, have this "*elongation arrested*," it will not become a cyme, but return to the state of a raceme, or rather of a spike; and, if by *all* the ramifications be meant the general peduncle as well as the partial ones, by such an arrest of their elongation, the panicle would not only assume the appearance of, but in fact would be, an umbel; if not a capitulum, or head. We use these terms in their improved but ordinary acceptations, without reference to the theory of Rœper, which, however ingenious it may be, our author, as well as ourselves, may be excused for not immediately, and in all its parts, adopting.

P. 347. "The style is frequently absent, and is no more essential to a pistillum than a petiole to a leaf, or a filament to an anther." True; but this sentence would appear to involve a false principle of physiology; for, although the filament of the stamen may be considered as analogous to the petiole of the leaf, the style of the pistil is the metamorphosis of a different part, viz. the elongated midrib; the ovarium is, in the scale of metamorphosis, the equivalent to the expansion of a leaf, and the peduncle (or the stalked carpellum, when it occurs,) afford examples of the various modifications of the petiole: hence the lengthened axis or midrib which, in the leaf, is often carried on in the form of tendril, should be likened to the style, of which it is the forerunner, in this series of transformations.

Par. 471. "As all seeds are matured ovula, and *all ovula* are originally enclosed within an ovarium, it is obvious that *naked seeds* cannot exist." Against this we immediately set, as a marginal note, ?! But the contents of the three following paragraphs somewhat lessened the astonishment we felt on the perusal of No. 471; for three modifications of naked seeds are known to exist: 1st, where the scales never assume the true form of pericarp, and the seeds are always and absolutely naked; 2d, where the pericarp, although present, does not close to include the seeds; and, 3d, where the seeds, at first enveloped, grow more rapidly than does the pericarp, which becoming ruptured, the seeds thenceforward remain truly naked? It is a general, not a universal rule, and should so have been stated. The *bull*, however, we suppose, was levelled at the Gymnospermious order of Linnæus; for the seeds of the labiate plants are, it is confessed, although apparently, not truly naked; and yet their pericarps are so thin and so tightly stretched, that, like the web dresses of the opera figurantes, they seem designed to exhibit what they pretend to conceal.

There are some other matters of minor importance, to which the author will do well to direct his attention, whenever a second edition of these Outlines may be wanted; for, notwithstanding the few imperfections we have thought it our duty to point out, we doubt not the book will be found a very useful manual for students, and as such we recommend it to their perusal.

Of the Synopsis, although it professes more, we think much less favorably: the design is bad, the execution worse. But we will not anticipate: every man has a right to state the theme of his discussion in his own words. An extract from our author's preface shall, therefore, introduce his volume.

"In submitting this little book to the public, it is right that something should be said in explanation of the reasons that have led to its preparation; especially as it may to some appear unnecessary, after the many useful, and in several respects very excellent, works that have been already published in illustration of the Flora of Great Britain. These have *all*, with the exception of the Flora Scotica of Dr. Hooker, been arranged upon the principles of a system which, whatever popularity it may, from particular circumstances, have acquired, and however useful it may have been found, in communicating a knowledge of the names of things, does certainly not now tend to the advancement of science, or to an accurate knowledge of things themselves.

"Of course, I allude to the *system of Linnæus*; a system which has almost disappeared from every country but our own, and *which ought now to find no other place in science, than among the records of things whose fame has passed away.* Hence *all* our British Floras are, in this view of the case, defective, with the exception* already made. I might therefore, without impropriety, stop at this point of my explanation; but, in addition *to the fundamental error* now adverted to, there is another of even more importance: the technical language in which these works are written is far from accurate; terms are applied in them vaguely and erroneously, and they so abound with mistakes, most of which are at variance with all correct notions of the structure of plants, that they are totally unfit to be placed in the hands of students. To these observations, the Flora Scotica of Dr. Hooker, and the Flora Edinensis of Dr. Greville, are honourable exceptions."

Upon the principles here laid down we are compelled to join issue both with Lindley and with Gray, especially with the former, who seems to have overlooked, or at least not duly estimated, the difference which truly does exist, and which ought always to be kept in view, between the objects of the so called artificial and natural schemes. Of the *so called,* we repeat; for neither of them are purely what they profess to be, neither of them are in truth what their appellations would denote: the artificial system has striven, yet in vain, to become natural, and the natural as vainly strives to combine with its synthetic powers, the analytical precision of its compeer, thus needlessly forcing itself to be, in many respects, artificial. Better would it have been for both, had each restrained itself to its respective province, and not, by endeavouring to supersede the necessity of an ally, manifested its insufficiency where it had no occasion to be ineffective, and rendered its exertions in its appropriate sphere much less energetic, and of much less worth.

Another object proposed to be accomplished in this work is "to reduce the language in which plants are described to a uniform standard, in correspondence with the purest principles of the science, but divested of unnecessary technicalities: and however far we may dissent from the former opinions expressed by the learned Professor, this, we must

* Why was not the Introduction by Gray added to this list of exceptions? It is arranged on the principles of the natural method, uses to excess the modern nomenclature, and was published in the year 1821. Surely Lindley's harbinger scarcely deserved to have his two thick volumes thus passed over without even a nominal allusion.

agree with him, is a desideratum in botany, and a labour fully worthy of his highest ambition. Hence we confess we were disappointed to find that, even on the titlepage, terms are used as synonymes which ought to express different and sometimes contrary meanings. Thus, the present volume is described as containing the "Vasculares, *or* flowering plants;" whereas, many of the vascular plants, e. g. the ferns and their allies, are destitute of flowers: they are thus excluded by the one term and included by the other; and shortly after we are advertised that "Cellulares, OR flowerless plants, are likewise terms that are used as synonymes. Now, whatever may be said of the mosses, which many botanists believe to have flowers, although they are cellular plants, no one can doubt that the ferns are both vascular and flowerless; nor is this contradiction in terms avoided by defining the vascular plants to be those which have *spiral* vessels, and the cellular (even when vascular) to be devoid thereof; for, passing the impropriety of the term, we have already found that spiral vessels have been described, by Sprengel and others, as existing in the inferior tribes of vegetables, such as the mosses, confervæ, &c. Gray very properly associated the ferns with other endogenous plants, to which their structure and mode of growth show a very strong alliance. But, even granting (which we do not) that the definitions are correct, still distinctions so recondite that botanists the most astute differ in their opinions concerning them, can never be esteemed fit or convenient diagnoses for students, or for general and common use.

The subdivision of the Vasculares, or flowering plants, into Dicotyledones and Monocotyledones, is liable to the same objection; for some of the first not only have more than two, but some of the Monocotyledones have two, others perhaps none; and, in many, the part usually taken for the cotyledon, as in the grasses, is no cotyledon at all. The mosses also have been said to have many cotyledons, although amongst the acotyledonous vegetables; but these parts have lately been called by some pseudo-cotyledons. Neither do the cylindrical and conical form, the branching disposition, nor the concentric structure of the stems, any more than the characters drawn from the reticulated or parallel veins of the leaves, universally hold good: all of them fail; in some examples they are absent, and in others the characters are so obscure as not to be relied on; e. g. Pinus, Cycas, Nymphæa, Cyamus, Hydrocharis, &c.

It is not against these distinctions that we contend, but against their use to the *exclusion* of a more certain and familiar index; an index which we cannot bring ourselves to think "ought now to find no other place in science, than among the records of things whose fame has passed away:" for, in almost every page of the "Synopsis" a like indeterminateness prevails. Thus, the Dicotyledones furnish three divisions, the Di- the Mono- and the A- chlamydeæ, which are described as having, in the first, "Calyx and corolla *both* present; occasionally imbricated and confounded with each other;" which latter part of the definition immediately confounds them with the second, which are said to have the "calyx only present, corolla none," as they are, by the occasional absence of both, confounded with the third. Thus, frequently among the Dichlamydeous plants, only one envelope is found, and occasionally none is present. Some Monochlamydeous plants have neither calyx nor corolla; and some Achlamydeous ones have more apparent calyx than that group of plants which are especially to be distinguished by its presence; e. g. Fagus, Euphorbia, Fraxinus, &c.

We have stated not only that the groups associated in the natural methods, even granting that they are natural, which they frequently are not, have far too discursive characters to be useful as an index; but also that the artificial indexes appended to the so-called natural orders, are less convenient, and far less perspicuous, than the Linnæan artificial system, which for them has been rejected, and is by some denounced as obsolete. It is necessary, therefore, that we justify this assertion by some examples: they might be taken from either of the works placed at the head of this notice, or from almost any other which has been published on a similar plan. We shall select the chief from Lindley's, because, as before stated, his being the most modern work, may be presumed to be the most improved, and, from his official situation, is the most likely to be influential in the botanic world.

Among the Exogenous, Dichlamydeous plants, we find the Ranunculaceæ, which order is defined as having sepals 3-6, petals 5-15; whereas, Clematis, the first genus, is stated to have petals *none*, or shorter than the sepals. Hooker says that the Clematis has no corolla at all. The second genus, Thalictrum, is likewise as obscure; for in it we are told that the sepals and petals are undistinguishable. In Caltha, also, and several other ranunculaceous plants, we read that the sepals and petals are "undistinguishable from each other," or "that they cannot be distinguished;" and yet upon the presence of both calyx and corolla (which are undistinguishable) depends, according to this analysis, the very circumstance of their being ranunculaceous plants. We are far from admitting that, in some of these cases, they are undistinguishable from each other; but that they are difficult of distinction, may be inferred from the circumstance of Gray and Hooker declaring that Clematis, Anemone, and Thalictrum, have no petals, and Smith stating that they have no calyx; one party considering the organs as sepals, the other as petals, yet both acknowledging that but one envelope is present. While Lindley, as before noticed, although he contends for their combined presence, admits that they are *undistinguishable;* and that they are truly so to him, may be inferred from the circumstance that, although he undertakes to revise* the phraseology of Smith, he calls the same parts sometimes calyx, and sometimes corolla; e. g. The genus Helleborus, defined as having a "calyx persistent of five sepals, and petals eight or ten, very short;" and the distinctions between the H. viridis and H. fœtidus are thus given: that in one the *petals* are spreading, in the other the petals are converging; whereas, the petals neither converge nor spread in either, but it is the sepals which do so. The organs which Smith has called (perhaps improperly) nectaries, Lindley chooses to call (perhaps not always more correctly) petals; but, then, what are petals in the generic definition, should also be petals in the specific, and not, as we find them in the genera Helleborus and Aconitum, sepals in the one and petals in the other; e. g. "Aconitum, calyx petaloid;—the upper *sepal* concave and helmet-shaped," &c. A. vulgare, "Upper *petal* arched at the back." Both references, as was the case with Helleborus, being to the same organ.

The Cruciferæ, one of the least exceptionable orders of the natural system, and one that has been the longest and most generally known, as it contains exactly the same plants that are arranged in the Linnæan artificial class Tetradynamia, affords a good instance for comparing the relative perspicuity of the two methods of analysis; and to its examination we unhesitatingly recommend our readers. It may also be permitted us to observe, that, although the Linnæan system be rejected, that here, as frequently elsewhere, the Linnæan diagnosis is resorted to; for, in the analytical table, the tetradynamious character of the stamens is set down as the essential diagnosis. Thus

Vasculares,

Exogynæ, or Dicotyledones,

Dichlamydeæ.

§Polypetalous.

*Stamens hypogynous, &c.

†Ovaria in more than one row, &c.

* "I have generally adopted its (i. e. the English Flora of Sir James Smith,) specific characters, the phraseology in which they were expressed having been carefully revised." Vide Preface.

††Ovarium solitary, &c.
Disk large,
—— small.
Sepals 2, deciduous.
* * * *
Sepals several,
Stamens tetradynamous, Cruciferæ,
—— not tetradynamous, &c.

Thus, after so many previous considerations, the Linnæan sign is at last resorted to as the most perspicuous, when it might just as well have been at first referred to.

The characters on which the suborders of the Cruciferæ are founded, although important to be known in the physiological history of plants, are any thing but convenient or perspicuous as diagnostic signs: e.g. instead of the Siliculosa and Siliquosa of Linnæus, we find the Pleurorhizæ, Notorhizæ, Orthoploceæ, and Diplecolobe; terms, the distinction of which are derived from the relative positions of the radicle and cotyledons, and the duplication of these latter; thus,

Radicle applied to the edges of the cotyledons, O = accumbent, 1
applied to the back of the cotyledons, O || incumbent,
Cotyledons straight, O || . . . . . . . . 2
folded lengthwise, O 7 7 . . . . . 3
doubled twice transversely, O || || || . 4

That these characters are not easy of application, may be inferred not only from the minuteness of the organs to be examined, and the difficulty of determining the fact with any but fully-formed seeds, but also from the circumstance that Mr. Robert Brown, undoubtedly one of the most talented botanists of the age, and to whose researches we owe these distinctions (physiologically important, although not systematically convenient,) at first believed that Subularia had the cotyledons doubled twice transversely; and as such it is arranged by Lindley in the sub-order Diplecolobeæ: whereas, they are not so folded, and consequently it ought not to be placed in that subdivision.

In the definition of the Caryophylleæ, it is stated that the "stamens are twice as many as the petals;" whereas, Mœnchia (the old Sagina erecta,) is defined as having "petals four, and stamens four." Sagina procumbens and Sagina apetala (the true S. minuta,) have also petals four; and stamens four; while Sagina maritima (the true S. apetela,) although a dichlamydeous plant, has petals none. Buffonia tenuifolia, or as De Candolle calls it, B. annua, like the other examples taken from this order, which is defined to have the stamens *twice as many* as the petals, has them, like Sagina and Mœnchia, *equal*, e. g. petals four, stamens four. Perhaps Cerastium semidecandrium, with its five stamens and five petals, is scarcely worth adding to the list of exceptions; but it must be remarked that, although the number of Linnæan exceptions has been sometimes exultingly referred to, that there is no convenient mode of reference here, as in the Linnæan artificial system, by which the nonconforming species are associated with their regular allies.

Again, the Rhamneæ are to be known by their stamens being opposite to the petals; whereas, the petals in the Rhamnus, which is notwithstanding a dichlamydeous plant, are often obsolete.

The definition of the Rosaceæ is, like that of many other orders,* very loose and comprehensive, and yet not sufficiently so to include all the plants that are arranged therein. Thus, calyx 4 or 5 lobed, petals 5, perigynous, equal, stamens *either* definite *or* indefinite; ovaries superior, *either* solitary *or* several; 1 celled; ovula 2 *or* more, suspended, very rarely erect; fruit *either* 1 seeded, nuts, *or* drupes, *or* follicles containing several seeds. Herbaceous plants *or* trees, (shrubs should also have been added;) leaves simple *or* compound, &c. And yet, among the plants included in this five-petaled order, the five petals being almost the only

* Thus, of Order 40 we read, "Calyx superior, usually with two *or* more bracteæ at its base, entire *or* lobed. Corolla superior, monopetalous, *or* polypetalous, rotate *or* tubular, regular *or* irregular. Ovarium with from one to five cells, one of which is often monospermous, the others polyspermous. Fruit indehiscent; one *or* more celled, *either* dry, fleshy, *or* succulent; seeds *either* solitary and pendulous, *or* numerous and attached to the axis; shrubs *or* herbaceous plants, with opposite *or* alternate leaves, &c.

In the same way Order 56, the Apocyneæ, described as "trees or shrubs" with "leaves opposite, sometimes whorled, sometimes scattered," having their "fruit follicular, capsular, drupaceous *or* berried, double *or* single." Orders 11, 12, 28, 31, &c. may also be referred to. Of the last it is said, "Petals 5, or by abortion 4, 3, 2, 1, *or* none, inserted into the base of the calyx, *either* papilionaceous *or* regularly spreading," &c.; stamens definite *or* indefinite, perigynous, *either* distinct, monadelphous, *or* diadelphous; very seldom triadelphous. Fruit *either* a legume *or* a drupa; seeds solitary *or* several, occasionally with an arillus; embryo destitute of albumen, *either* straight *or* with the radicle bent upon the cotyledons; cotyledons *either* remaining underground in germination, *or* elevated above the ground and becoming green leaves." And Order 25, Saxifrageæ, runs thus, "Calyx *either* superior *or* inferior, of 4 *or* 5 sepals, which cohere more *or* less at their base. Petals 5 *or* none, inserted between the lobes of the calyx. Stamens 5, 10, inserted *either* into the calyx (perigynous,) *or* beneath the ovarium (hypogynous), &c. Disk *either* hypogynous *or* perigynous, sometimes nearly obsolete, sometimes annular and notched, rarely consisting of 5 scales. Ovarium adhering to the calyx *or* distinct from it; usually consisting of two parts, cohering more *or* less by their face [quære, base?] but distinct at the apex; sometimes 2 celled, with a central placenta; sometimes 1 celled, with parietal placentæ; rarely 4 *or* 5 celled, &c. Fruit generally a membranous 1 *or* 2 celled capsule, with two bracteæ; rarely a 4-celled, 4-valved capsule, sometimes a 4 celled berry," &c.

character given absolutely, no character varies more from accidental circumstances, even in the normal genus Rosa.

In Dryas octopetala, the petals are, as its name imports, even when growing spontaneously, eight or more; whilst in Tormentilla, another of these five-petaled rosaceous plants, they are only four. In Sanguisorbeæ, also, when present, for there are often none at all, (the analytical table says "petals none,") they are only four; and in the genus Sanguisorba they are altogether absent. Alchemilla and Poterium likewise are defined by Lindley as being destitute of corolla, and yet they are analytically arranged among the pentapetalous Dichlamydeæ. We find that, following continental authors, the genus Tormentilla is, both by Gray and Lindley, merged in the genus Potentilla: against this, however, we must protest, on what authority soever it may be attempted. Potentilla is defined, in the Synopsis, as having five petals; Tormentilla, one of its would-be Lindlean species, has only four. Again, the two old species of British Tormentilla were named T. erecta and T. reptans, which are set down in the Synopsis under the names of Potentilla Tormentilla, and P. reptans, without reflecting that there was already a P. reptans; so that, as they now stand, we find in the Synopsis two very different plants both bearing the same appellation. This is but a solitary instance of the careless manner (we can attribute it to nothing else,) in which this compilation has been made: many others might be easily pointed out which but ill accord with the profession in the preface. Even Gray, who likewise joined the two genera, avoided it by giving to Tormentilla reptans the name of Potentilla nemoralis, and to T. erecta the name of Potentilla officinalis.

The Halorageæ likewise are placed between the Circæaceæ and Umbelliferæ, all in the polypetalous section of the dichlamydeous Dicotyledons, and the only two genera enumerated are Myriophyllum and Hippuris, the first of which has no petals in the female flowers, and the latter none at all; and the genus Œnanthe, one of the Umbelliferæ, is defined to have the "universal involucrum wanting," while, in the face of this generic diagnosis, one of its species, Œnanthe pimpinelloides, is immediately afterwards said to be distinguished from the others by having the "universal involucrum many-leaved."

As, among the polypetalous plants, we find some which have all the petals soldered into one, or all become obsolete save one, or in some cases none left at all, thus varying from 5 to 4, 3, 2, 1, or 0; so, among the monopetalous plants, we find the Caprifoliaceæ to be both mono- and poly- petalous: i. e. Lonicera has one petal, Cornus four, and Hedera five: but of this enough; for we are compelled, from consideration both of space and time, to pass over many of the orders with a very cursory examination. Still our attention becomes immediately arrested by a peculiar physiological discovery, hinted at by the division of Anthemis cotula, under the name of Maruta fœtida, from the other species of Anthemis; for this new genus is said to have the flowers radiant; of the *disk* NEUTER, of the *ray* FEMALE. How the female florets of the ray become fertilised by the *neuter* florets of the disk, we must leave the learned Professor to explain: we freely confess that the subject passes our humble understanding; the only guess that we can venture is, that the pistils of some neighbouring Anthemis kindly consent to lend their potential stamens to the disconsolate Maruta, who would seem else condemned for life, as Darwin would have written, to the soft attentions of platonic love. However, the central florets of Anthemis cotula (or Maruta, if it must be so,) are in truth bisexual instead of neuter, and those of the ray are female; and thus we find, on reference, they were described by Cassini, to whom the change of the genus is attributed; so that, instead of having no seeds at all fertilized, the whole become perfected, and consequently the plant will stand in the order Polygamia superflua of Linnæus's Syngenesia. Was it not from the Linnæan nineteenth class that the "connate anthers" were taken, as the most important analytical character (vide table, page 6,) of the Compositæ? all of which are defined as having "the anthers cohering into a cylinder." But are not the anthers of Xanthium distinct? and yet it is enumerated amongst the syngenesious plants, and stands between Achillea and Bidens, without even a hint as to this peculiarity of its structure, which Linnæus noted by excluding it from his nineteenth class. In Tussilago hybrida, likewise, the anthers scarcely, if at all, coalesce.

The Plumbagineæ, which stand among the dichlamydeous monopetalous plants, have their corollæ both "monopetalous and polypetalous; and the Oleineæ, still dichlamydeous plants, and defined as having the calyx monophyllus and the corolla *monopetalous*, and occasionally of *four petals*, include Fraxinus, which genus is, in the first sentence of its definition, thus described: "*Calyx and corolla none*," &c. Order 54, Ericeæ, is said to have the "fruit *capsular*;" and, immediately under this definition, we find an analytical table of the order, in which the genera

Arbutus and Arctostaphylos* (we hate these new and useless names,) are distinguished from their associates by bearing berries.

The Gentianeæ, with the same carelessness, have been said to have leaves opposite," and then immediately the order is subdivided into two sections or sub-orders, of which one is distinguished from the other by having the leaves *alternate*. In the analytical table, page 7, Orders 54 and 55 are distinguished from Orders 56, 57, and 58, thus,

"Anthers dehiscing by *pores*, Ericeæ, &c.
"Anthers dehiscing by *valves*, Solaneæ;" &c.

but, on turning to page 182, we find that Solanum, the very genus that gives name to the order, is defined to have "anthers oblong, opening by two *pores* at the apex." 59, Primulaceæ, a dichlamydeous order, is thus defined, "Calyx divided, five cleft; corolla monopetalous, &c. The third genus, Glaux, is thus defined, "Calyx campanulate, corolla *none*," &c. The Scrophularineæ are described as having opposite leaves, (p. 187;) and three species of the genus Linaria are distinguished by having their leaves either wholly or chiefly alternate, (p. 191.) Digitalis, likewise, one of the most important plants in the order, has alternate leaves. 224, Empetrum, put among the Monochlamydeæ, is described by Smith as having both "calyx and corolla;" and of the Euphorbiaceæ, all of which are classed among the Monochlamydeæ, we find (page 228) this pertinent observation.

*Note*. "The genus Euphorbia, among Monochlamydeæ, being *destitute of calyx and corolla*, may, by the student, be referred to some order of this division; but in that genus the absence of floral envelopes is to be ascribed to the excessive developement of the involucrum; the other genera of the same order are furnished with calyx."

To whatever cause the absence of calyx and corolla may be attributed, when such characters are taken for an index, plants destitute of both should be put amongst the Achlamydeæ, especially in an analytical work designed, as this professes to be, for students; for no notice of this is taken in the analytical table, page 5, by which the first step of the

* We scarcely think that the introduction of this, and many other such-like new subgeneric names, can be in consonance with the declaration in the preface, which states that "such discretion has been exercised as has protected the rights of British botanists, wherever they have deserved protection; e. g. Catopodium, Diplotaxis, Maruta, Silybum, Trichonema, Chondrilla, Capsella, cum multis aliis.

investigation is to be conducted, and by which it is supposed that "the subject has been so far simplified that as great a facility is given to acquiring a knowledge of botany as can be offered even by the Linnæan system;" for by it students are to find out unknown plants, and for it they are to reject the Linnæan clue. This note concludes by observing that "there is a tendency to produce a calyx in Cupuliferæ;" and hence, amongst the achlamydeous plants, we find that Fagus is said to have a six-lobed calyx.

Of Order 82, Coniferæ, we read "stamens *distinct*;" and of Taxus, in this order, it is said, "stamens *eight or ten monadelphous*." The account of the fruit of the Coniferæ seems to be likewise very incomplete, if not confused. Order 84, Callitrichineæ, even if separated from Order 37, Halorageæ, which to us appears a work of supererogation, should never have been so widely severed. Myriophyllum and Hippuris, as before observed, have no right to hold the rank in which they now are found.

Hurried as have been the observations which our marginal notes on the Dicotyledons have compelled us to make, those on the Monocotyledons must be still more brief; yet, in justice to ourselves, and in consideration to our author, we must give him a few more hints for the improvement of the second edition of his Synopsis.

Of the sub-class ii. Monocotyledones, it is written, "Leaves generally sheathing at the base, and not articulated with the stem, ALWAYS *alternate*, with parallel simple veins traversing the space between them." Potamogeton densus has its *leaves opposite*; and Hydrocharis, with several of the Aroideæ, have the veins of the leaves, at any rate apparently, reticulated. The order Smilaceæ, in the analytical table, p. 245, is arranged along with those having a superior ovarium, while a note* confesses what every tyro knows, that Tamus, one of the Smilaceæ, has the ovarium inferior. This 98th order, page 270, is furthermore defined as having "Perianthium *inferior*, petaloid, six parted, stamens *six*, ovary *three*-celled, stigmas *three*." But if we examine how the genera agree with the characters of the order in which they are placed, we shall find that several are dissenters: thus, Ruscus has but one stigma, and Paris

* Why not have put inferior *or* superior in the definition at once; there are plenty of precedents in the volume; that at page 85 would have kept it in countenance. Aroideæ, "stamens definite *or* indefinite; fruit succulent *or* dry; seeds solitary *or* numerous." Herbaceous plants *or* shrubs, stemless *or* arborescent, *or* climbing by means of aerial roots; leaves *either* with parallel *or* branching veins, often cordate, &c."

differs still more widely, having eight stamens, four stigmas, four sepals, four petals, and its berry is four-celled.

In the analysis of the orders, we find that some of the Petaloideæ are distinguished from the others by having *no petals*; and that the two orders which have the "Perianthium wanting" are inter-distinguished by their having respectively their "stems *leafy*" and their "stems *leafless*." Those with the leafless stems form the 88th natural order, Pistiaceæ; and, on turning to page 251, where it is defined more at length, it is said that it contains "floating minute plants, with very vascular, lenticular, or lobed *stems*, LEAVES NONE; flowers appearing from the margins of the stems." The only British genus in this order is the duckweed (Lemna), and in the generic definition the dogma is a third time repeated, that these plants have "no leaves."

The genus contains four species, which are thus inter-distinguished:

"L. trisulca, *leaves* stalked, elliptic, lanceolate, proliferous.

L. minor, *leaves* obovate, flattish above and beneath.

L. gibba, *leaves* obovate, slightly convex above; hemispherical beneath; roots solitary.

L. polyrrhiza, *leaves* roundish, obovate, convex beneath; roots clustered."

Thus we find that four species, all of which have *leaves*, compose a genus that is *leafless*.

Here must our observations for the present cease, and, slightly as we glanced over the dicotyledonous section of the Synopsis, our marginal notes were so numerous, that selecting but a part of it has swelled our review so much, that the Monocotyledones have been necessarily still more lightly touched on. Some whole families, and those important ones, have hardly, or not at all, been noticed, viz. the Compositæ, Umbellatæ, Gramineæ, &c.: these, therefore, we recommend to Mr. Lindley's more especial consideration; for, notwithstanding all its defects, his Synopsis is a valuable work, very much simpler than any other we have met with, and divested in a great measure of those very useless and discordant technicalities that bewilder and disgust the student, without benefiting in any degree the science; and had it been proposed as a conspectus of the natural orders, or as a praxis for the British student, in furtherance of, not as a substitute for, the Linnæan system, most of the objections to the looseness of the definitions would have been cancelled, for then they would have been taken for what they are worth, for what in truth they are, descriptions rather than diagnoses. But when the author declares, "Above all things I have laboured to remove the difficulties that at present attend the study of the natural affinities of plants, both in this country and elsewhere;" when he states that "for this purpose (he has) prefixed to each class and order analytical tables of their contents, in which tables the most important characters are employed to distinguish one thing from another, and the less important peculiarities are kept out of sight;" and when, as the result of his labour, he hopes that, by such means, the subject will be found simplified, and that "as great a facility will have been given to acquiring an incipient knowledge of botany as can be offered even by the Linnæan system," we are bound to tell him that on this point his labour has been wholly in vain. Let him cast his eye over the analytical table, and say whether, to a student, the abundance or absence of albumen in the Oxalideæ and Geraniaceæ, the embryo curved or generally straight in the Paronychiæ, Saxifrageæ, &c., the dehiscence of the anthers, the cotyledons rising above or remaining beneath the ground, the erect or pendulous ovula, the erect or inverted embryo, the glands of the pollen enclosed or naked, the pollen in two or four masses, &c. are *obvious* diagnostic signs? and again, although we must agree (physiologically, not systematically,) with our author, that, "upon the niceties by which the genera of many orders, such as Gramineæ and Orchideæ, are distinguished, some of the most curious parts of botany will be found to depend," still we must contend that such niceties are not *obvious* characters: and this we think that he will himself be ready to confess, if he turn to the list of Corrigenda which, a year after its publication, it was found necessary to append to the volume, and in which several of such characters are erased. Indeed, we procured this page of Corrigenda before we ventured to commence the present task; and, although it has relieved us from many of our difficulties, by the corrections it has introduced, it has, from not being published till so long after the work, but served to confirm more strongly the appropriation of those misappliances which are left unchecked, and to which alone our observations have been attached; for we have expunged all comments on those errors which the author has himself observed, and thus subsequently confessed. Neither can we agree with the doctrine, that "whatever the difficulties may be of becoming acquainted with plants, according to this method, they are inseparable from botany, which cannot be usefully studied without encountering them:" for the Linnæan system, we contend, does separate from a very useful part of botany much of the difficulty in which, by the natural arrangement, it is needlessly involved. "A mineralogist (he continues,) may as well complain of the necessity of a blowpipe, or a chemist of the infinite variety of apparatus which he is compelled to employ, as a botanist of the microscope and dissecting knife." True; it is not of the use but of the abuse of the microscope and dissecting knife that a botanist has a right to complain. An anatomist has no right to complain of the scalpel when he wishes to investigate structure; but it would be ridiculous to compel him to dissect an animal, and ascertain whether the lungs be entire or pierced, before he must call it a bird or a beast, or to open the abdomen of a quadruped to search for the gall-bladder, before he must decide whether it be a horse or not, when the hoofs, the teeth, and other external obvious characters, will more conveniently decide that question. The astronomer royal has no right to complain of the use of a telescope, when he wants to survey the satellites of Jupiter, but it would be ridiculous to compel him to contemplate Blackheath and the park through the instrument alone, when his unassisted eyes will ensure him a better prospect. "It would undoubtedly be more convenient (adds our author,) if knowledge could be acquired with greater facility; but we must take things as we find them, and submit patiently to the difficulties of the road we are forced to pursue." Of a truth; but why *make* difficulties which are foreign to the route? why forsake a straight and easy, for a winding and arduous road? It is confessed that the Southron has no right to complain of the distance between London and Edinburgh; but it would be ridiculous to compel the inhabitants of Glasgow to travel, *via* London, to the Orkneys.

Linnæans, (perhaps erring, but not prejudiced Linnæans,) as we confess ourselves to be, it has been with pleasure, not with jealousy, that we have watched the labours of those who seem to consider themselves as our opponents; convinced that, although not with us, they are for us, as no researches after truth can ever be against us; convinced that the more investigators can be induced to enter this field of inquiry, the more various their objects, and the more different their routes, the more speedily will truth be found, and the more rapid will be the advance of knowledge. At the same time, we are convinced that the pre-eminent success and brilliant fame of Linnæus has oft-times led his followers astray; led them to devote themselves too exclusively to the denomination and classification of plants, to the comparative neglect of the more interesting and philosophic branches of botany, which were never overlooked by their great master, viz. the intimate structure of plants and the laws of vegetable function.

Much as schisms are in general condemned, schisms in philosophy are eminently useful: they provoke inquiry, and they elicit truth. In no instance have they been more needed, in none have they been more valuable, than amongst the students of the vegetable world: a degree of indolence and apathy, a baneful reverence for doctrines merely because they were Linnæan, as if Linnæus had been any thing more than man; doctrines which were no longer tenable, and which he himself would have been the first to eschew, had he been acquainted with the discoveries of these latter days, had too long and too prevalently distinguished his disciples; and it required the energizing stimulus of sectarian proselytism to rouse them from that inertia which had naturally succeeded so brilliant a career. The Linnæan artificial scheme was never designed, was never fitted to become any thing more than an index to a natural Flora, and a most convenient, a most advantageous, index it has been: not that it is without its faults; it has its difficulties, and these we may take some future occasion to point out; but it is more simple, more perspicuous, and more familiar than any other that has ever been devised. The multitude of particulars which necessarily enter into the definitions of the natural orders of plants, for every organ should be investigated, and every peculiarity of structure noted, precludes their use as an index, as a finder: they are, in truth, rather descriptions than diagnoses. The machinery is too complex, far too cumbrous, for ordinary use; it forms, in truth, the subject matter of the science, and, to use it as a clue, is to consign it to a very degrading duty; it perverts the very benefits of the copious details to extremely injurious ends, and resembles the ridiculous attempt of transferring the substance of a volume to its table of contents or to the index at the end.

The late Professor Martyn once observed, that he had been "long convinced in his own mind that we strive in vain to unite a *natural* with an *artificial* arrangement;" yet this Utopian scheme has ever continued uppermost, to bewilder the exertions of systematic botanists, and hence method after method, and system after system, have been invented and declined; change after change, and reformation after reformation, have met with an equal fate. The reformers, or rather the mutilators, of the Linnæan scheme, have too generally succumbed to this philosophic prejudice; by the abolition of the order Monogamia of the nineteenth class, Syngenesia, because the flowers are not compound, as well as other changes, they have attempted to reconcile these antitheses. Even Linnæus himself, in some measure, sanctioned the error by his example; for there is no good reason why Lychnis dioica, Bryonia dioica, &c. should not have been put in the class Dioecia, and other nonconforming species suffered to stand with the artificial allies, which by their structure they had selected for themselves, as the object of the scheme is only to find them out: and, on the other hand, the advocates of the natural methods have too often endeavoured to reduce them to the form of catalogues, and some modern botanists now declare that the natural affinities of plants are so far known that the artificial clue may be dispensed with, and strenuously advocate the doctrine that the single mean is all-sufficient, both as an index and a history of plants. Indeed, one has not scrupled to assert that the system of Linnæus "ought now to find no other place in science than among the records of things whose fame has passed away;" but they have all been under the necessity of adding artificial clues, but which clues, as indexes, are, as we have shown, far less convenient and perspicuous than the Linnæan which they reject. Moreover, this natural synopsis itself, as far as it is an index, is confessed, even by the author, to be artificial; as artificial, and far less simple and perspicuous, than the Linnæan: so true is the observation in the preface, that the "tables are entirely artificial, and have not been constructed with reference to any thing beyond what is to be found in the present work (and very carelessly as to this); they are often not applicable to other works, (and, as we have shown, the disjunctive conjunction might also have been introduced here, and the sentence have been read "they are often not applicable *either* to other works *or* to this,") for they are a very inefficient "analysis of the characters of such genera and orders as this book contains."

Notwithstanding his denunciation of our favorite system, we take leave of Mr. Lindley with the most sincere good-will. He must forgive the strictures we have been compelled to make, and consider them, as indeed they are, the greatest compliment we had it in our power to pay him: other works, and by other pens, might have been slightly mentioned, and some there are unworthy of much comment. A person of less authority, and less favorably known to the philosophic world, could have done much less harm by any erroneous doctrines he might have chosen to promulgate; but a man of Mr. Lindley's standing and ability is likely to lead after him the third part of Britain. Therefore it became our duty to offer a few words of warning, explanation, and advice, which the present aspect of the botanical horizon seemed to render not unnecessary to those who, being about to commence their studies in this department of natural history and philosophy, may be led to cast in their lot with either of the two great parties which so unnecessarily and, in some respects, injuriously divide the botanic world. Neither may they be without their use to the intemperate of either party, if they should show the defenders of the so-called artificial system that a farther step in natural arrangement is perfectly compatible therewith; and more especially should they convince the advocates of the misnamed natural scheme, that, so far from the Linnæan system having become obsolete, it was never more needed than at the present day. For our part, we most firmly believe that the true value of the artificial will prove to be as an index to the natural orders, and that the natural method will never be practically useful without the assistance of the artificial key. Sentiments such as these were entertained by the great founder of the one, who likewise contributed much to the advancement of the other: and sentiments such as these would seem to have been adopted by many, if not most, of the more enlightened botanists who have flourished since. Few, if any, of the disciples of Linnæus have been blind to the imperfections of his arbitrary system; their researches, in truth, have furnished the most potent arms that its adversaries would turn against it. Yet Hudson, Martyn, Miller, Thunberg, Curtis, Withering, Aiton, Salisbury, Persoon, Smith, Sowerby, Sprengel, Hooker, and many others, though eminently versed both in practical and theoretic botany, were so fully convinced of the great utility of the Linnæan artificial scheme, that, never forgetting the superior interest of natural arrangement, they have employed themselves to perfect, not to overthrow, the one, before they could hail with certainty the advent of the other; and most of them have doubted the possibility of its double use.

Some botanists, however, have differently thought, and, pursuing their researches into the affinities of plants, have believed a clue discovered which would dispense entirely with the artificial scheme: and to this we in a great measure owe the recondite investigations into vegetable affinities, which have been prosecuted with laudable zeal both in this country and abroad, but more especially in France; and the French, who never cordially adopted the system of

Linnæus, have earnestly attempted to supersede its use by the much-improved Jussieuan arrangement. English botanists, less nostalgic than their neighbours, adopted the Swedish scheme, when persuaded of its value, and having laboured much and wrought successfully in its improvement, now hesitate to abandon what has proved so sure a guide, for a more speculatively beautiful, though less practically useful, system; for the observations of Linnæus seem as relevant to botany in the present age, as they were to its state as a science when he wrote.

"Multitudo generum est onus memoriæ, levandum systemate.

"Systema indigitabit absque præceptore plantam.

"Ordines naturales non constituunt methodum absque clave.

"Methodus artificialis itaque sola valet in diagnosi; cum clavis methodi naturalis vix ac ne vix possibilis sit.

"Ordines naturales valent de natura plantarum.

"Artificiales in diagnosi plantarum.

"Qui loco methodi naturalis disponunt plantas secundum ejus fragmenta respuuntque artificialem, videntur mihi iis similes, qui commodam et fornicatam domum evertunt, inque ejus locum re-ædificant aliam, sed tectum fornicis conficere non valent."

No words could more fully show the enlarged and liberal spirit of the philosopher, and thus, where his mantle has descended, it will be always found that the most devoted disciples of Linnæus are also to the natural mode the truest friends.

---

### COLLECTANEA.

Floriferis ut apes in saltibus omnia libant,
Omnia nos, itidem, depascimur aurea dicta.

